# Structural maturation of the HIV-1 RNA 5’ untranslated region by Pr55^Gag^ and its maturation products

**DOI:** 10.1080/15476286.2021.2021677

**Published:** 2022-01-22

**Authors:** Orian Gilmer, Elodie Mailler, Jean-Christophe Paillart, Assia Mouhand, Carine Tisné, Johnson Mak, Redmond P. Smyth, Roland Marquet, Valérie Vivet-Boudou

**Affiliations:** a Université de Strasbourg, CNRS, Architecture et Réactivité de l’ARN, UPR 9002, IBMC, Strasbourg, France; b Expression Génétique Microbienne, UMR 8261, CNRS, Université de Paris, Institut de Biologie Physico-chimique, Paris, France; c Institute for Glycomics, Griffith University, Gold Coast, Australia

**Keywords:** HIV-1, genomic RNA, RNA structure, maturation, Gag, Pr55^Gag^, NCp15, NCp9, NCp7, RNA chaperone

## Abstract

Maturation of the HIV-1 viral particles shortly after budding is required for infectivity. During this process, the Pr55^Gag^ precursor undergoes a cascade of proteolytic cleavages, and whilst the structural rearrangements of the viral proteins are well understood, the concomitant maturation of the genomic RNA (gRNA) structure is unexplored, despite evidence that it is required for infectivity. To get insight into this process, we systematically analysed the interactions between Pr55^Gag^ or its maturation products (NCp15, NCp9 and NCp7) and the 5ʹ gRNA region and their structural consequences, *in vitro*. We show that Pr55^Gag^ and its maturation products mostly bind at different RNA sites and with different contributions of their two zinc knuckle domains. Importantly, these proteins have different transient and permanent effects on the RNA structure, the late NCp9 and NCp7 inducing dramatic structural rearrangements. Altogether, our results reveal the distinct contributions of the different Pr55^Gag^ maturation products on the gRNA structural maturation.

## Introduction

Retroviruses, including human immunodeficiency virus type 1 (HIV-1), bud from infected cells as immature non-infectious particles [1]. Shortly after budding of HIV-1 particles, proteolytic cleavage of the Pr55^Gag^ and Pr160^GagPol^ precursors triggers morphological rearrangements of the immature Gag shell and lead to formation of the cone-shaped capsid characteristic of the lentivirus family [1–4]. At the same time, the genomic RNA (gRNA) rearranges from a ‘loose’ unstable dimer to a more stable and compact ‘tight’ dimer [5,6]. This process is broadly known as ‘maturation’ and is required for the formation of an infectious particle. The proteolytic cleavages taking place during maturation are well characterized [1,3,7,8]. Indeed, the tridimensional structures of the HIV-1 capsid (CA) in immature [9] and mature [10] virions, as well as in mutants blocked at different stages of maturation have been solved by cryo-electron microscopy [11], revealing the structural switch triggering CA maturation.

**Figure 1.**
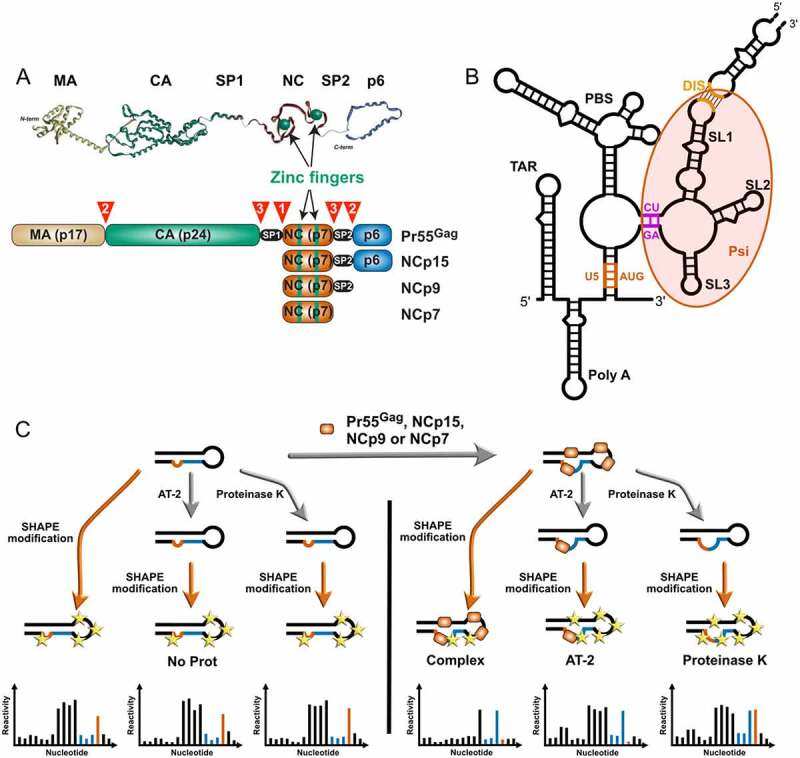
Players and experimental strategy. (a) The Pr55^Gag^ precursor and its nucleocapsid-containing maturation products. Scheme of the Pr55^Gag^, NCp15, NCp9, and NCp7 proteins are drawn in the lower part to indicate the matrix (MA), capsid (CA), spacer peptide 1 (SP1), nucleocapsid (NC), spacer peptide 2 (SP2) and p6 domains. The sequential cleavages of the Pr55^Gag^ precursor are indicated by numbered red arrowheads and the zinc fingers located in the NC domain are indicated in green. The 3D structures of the individual domains are shown on top of the figure and these structures are artificially linked together in a linear manner as no 3D structure of the full-length Pr55^Gag^ is available. (b) Secondary structure of the 5ʹ region of the HIV-1 genomic RNA (gRNA). One of the secondary structure models proposed in the literature is drawn to indicate the main elements present in this region, namely from 5ʹ to 3ʹ: TAR, the *trans*-activating region of gRNA transcription; polyA, which contains the repressed 5ʹ copy of the polyadenylation signal in its apical loop; U5:AUG (coloured in Orange), a proposed long-distance interaction between the U5 (unique in 5ʹ) region and the region surrounding the AUG initiation codon of the *gag* gene; PBS, the primer binding site domain to which tRNA^Lys,3^ has to be annealed to initiate reverse transcription; CU:GA (coloured in purple) a proposed long distance interaction between CU- and GA-rich regions; SL1, stem-loop 1 which contains the gRNA dimerization initiation site and is involved in gRNA packaging; SL2, which contains the main 5ʹ splice site; SL3, which is also involved in gRNA packaging. While most of these elements are present in the majority of secondary structure models proposed in the literature, the sequences forming the U5:AUG and CU:GA long distance interactions may be involved in alternative interactions. (c) Experimental strategy used in this study. gRNA 1–600 was refolded *in vitro* and incubated with Pr55^Gag^, NCp15, NCp9 or NCp7 protein (right). SHAPE was performed directly on the RNA:protein complex (***Complex*** condition) or after treatment of the complex with AT-2 (***AT-2*** condition), which is able to eject Zn^2+^ ions from the two zinc fingers located in the NC domain. Alternatively, the protein was removed by proteinase K before performing SHAPE (***ProtK*** condition). Controls without protein were perform for each of the three conditions (left). However, no significant differences were observed between these three controls, which were thus pooled together (***NoProt*** condition).

Proteolytic processing of Pr55^Gag^ is driven by ordered, sequential cleavages at five positions by the viral protease enzyme [1,3,7,8] (Figure 1(a)). Correct proteolytic processing of Pr55^Gag^ is necessary not only to form the mature matrix and capsid [1–3], but also for generating the mature gRNA dimer [5,6,12]. As the nucleocapsid (NC) domain exhibits potent RNA chaperone activity, interaction of Pr55^Gag^ and its NC-containing maturation products with gRNA are expected to govern its structural rearrangements [13–15]. The primary cleavage between spacer peptide 1 (SP1) and the NC domain (Figure 1(a)) releases NCp15 inside the virion [6,12,16,17] and is associated with the initial condensation of the viral genome [18]. The secondary cleavage events, which release NCp9 (Figure 1(a)), have no further impact on gRNA compaction [19], but the late cleavage event that generates mature NCp7 (Figure 1(a)) is necessary to complete this process [20]. Intriguingly, mutant viruses blocked after the primary cleavage event contain the same level of reverse transcriptase activity as fully mature virions and are competent for virus-cell fusion, yet these viruses display a marked defect in infectivity that is correlated to a failure to accumulate late reverse transcription products [18,19]. Furthermore, morphological changes in the virion require neither the NC domain nor gRNA [21] and they do not necessarily match with maturation of the gRNA [16,18,22]. Thus, morphological maturation of the virus is not sufficient for infectivity but must be accompanied by maturation of the gRNA structure, and it has been reported that the native structure of the HIV-1 gRNA is required for successful reverse transcription [23,24].

However, the relationship between virion protein maturation and structural maturation of the gRNA is poorly understood, despite the fact that these two phenomena are intricately linked, as gRNA increases the kinetics of Pr55^Gag^ cleavage by the viral protease [8,25]. The structural rearrangements of the gRNA taking place during maturation remain largely unknown, mainly because it is extremely difficult to analyse the gRNA structure in virions blocked at different steps of the maturation process. Indeed, while recent progresses in chemical probing has made it possible to analyse the structure of viral RNAs in infected cells or within viral particles or after extraction, such studies are still restricted to infectious viruses [26–29]. As an alternative to such an approach, here, we systematically compared binding of Pr55^Gag^, NCp15, NCp9, and NCp7 to the 5ʹ untranslated region (5-UTR) of HIV-1 gRNA. We used RNA chemical probing to compare the protein footprints and their transient effects of the RNA structure, the importance of the zinc fingers in the binding of these proteins to gRNA, and the permanent refolding they impose to the gRNA structure. Indeed, while several Gag [30–33] and NCp7 [26,34,35] footprinting studies have been reported in the literature, no systematic comparison of Pr55^Gag^ and its consecutive maturation products, which would provide insight into the gRNA structural maturation process, is available.

We focused on the interaction of Pr55^Gag^ and its NC-containing maturation products with the 5ʹ-UTR of HIV-1 gRNA because this region is replete with sites that play key roles in HIV-1 replication and whose functions are directly linked to their structure (Figure 1(b)). Of special interest in the context of viral assembly and maturation are the primer binding site (PBS) domain and the packaging signal (Psi) region (Figure 1(b)). The PBS domain contains several sequences that are complementary to tRNA^Lys3^, which serves as a primer for HIV-1 reverse transcription, including the PBS itself, which is complementary to the 18 nucleotides (nts) at the 3ʹend of tRNA^Lys3,^ and other regions complementary to the tRNA^Lys3^ anticodon loop [36–38] and the TYC arm [39,40]. The Psi region corresponds to a ~ 110 nt long sequence that contains all the elements required for Pr55^Gag^ binding [41] and is crucial for gRNA selection and packaging [42]. This region can adopt several different conformations, which have been proposed to regulate the switch from translation to packaging of the full-length unspliced gRNA [43–45]. It contains the conserved stem-loops 1 to 3 (SL1 to SL3) [46,47]: SL1 and SL3 play major roles in gRNA packaging [42,48], whereas SL2 regulates splicing of the full-length transcript and prevents cleavage and polyadenylation at the 5ʹ copy of the polyadenylation site [42]. In addition, SL1 initiates dimerization of gRNA, which is mediated by a self-complementary sequence in the SL1 apical loop [49,50].

## Results

### Experimental strategy

In order to assess the capacity of Pr55^Gag^ and its maturation products NCp15, NCp9 and NCp7 to bind and rearrange the 5ʹ-end region of the HIV-1 genome, we performed detailed RNA structure and protein binding analysis using high-throughput selective 2ʹ-hydroxyl acylation analysed by primer extension (hSHAPE). SHAPE experiments interrogate RNA structural dynamics using electrophilic anhydride that preferentially acylate the 2ʹ-hydroxyl (2ʹ-OH) ribose groups of single stranded RNA [51,52]. 2ʹ-OH acetylation leads to a block to reverse transcription that can be quantitated using fluorescent oligonucleotides on a capillary electrophoresis device [26]. SHAPE experiments are routinely used to probe RNA structures [51–53] and protein binding sites in viral genomes [26,30,54,55]. Here, we performed all hSHAPE experiments on *in vitro* transcribed RNA corresponding to the first 600 nts of HIV-1 gRNA under well-defined experimental conditions. In our analysis, we focused on the untranslated region (nucleotides 1–335), as it contains functional sites that play key roles in HIV-1 replication (Figure 1(b)) and is evolutionary more conserved than the HIV-1 gRNA coding regions [47].

To test the effect of Pr55^Gag^ and its maturation products on the structure of the 1–600 gRNA, we analysed four conditions (Figure 1(c)): 1) gRNA 1–600 in the absence of protein (***NoProt*** condition), 2) gRNA 1–600 in complex with Pr55^Gag^, NCp15, NCp9, or NCp7 (***Complex*** condition), 3) gRNA 1–600:protein complexes treated with aldrithiol-2 (AT-2), a compound that is able to eject the Zn^2+^ ions from the two zinc fingers located in the NC domain [26] (***AT-2*** condition), and 4) gRNA 1–600:protein complexes treated with proteinase K (***ProtK*** condition). Since we wanted to mimic conditions prevailing in viral particles, and viral particles are known to contain between 1,000 and 5,000 of Pr55^Gag^ or its maturation products [56], all complexes were formed at a ratio of 1 protein molecule per 10 nts of gRNA 1–600. Comparing the averaged SHAPE reactivity values of the ***Complex*** condition with SHAPE reactivity values of the ***NoProt*** condition allowed us to identify the protein binding sites on gRNA 1–600, as well as the effects of protein binding on the RNA structure. The reverse footprints of the two zinc-fingers located in the NC domain of full-length Pr55^Gag^ and its maturation products (Figure 1(a)) were identified by comparing SHAPE reactivities of the ***Complex*** condition with the ones of the ***AT-2*** conditions. Finally, in order to visualize the permanent effect of the proteins on the RNA structure after they have been removed, *i.e*. their RNA chaperone activity, the SHAPE reactivities of the ***ProtK*** samples were compared to the ones of the ***NoProt*** samples.

Separate controls without protein were performed for each of the ***Complex, AT-2***, and ***ProtK*** conditions (Figure 1(c)**, left part**), but since they did not show any significant differences amongst conditions, all ***NoProt*** datasets were pooled together (**Supplementary Dataset 1**).

In order to minimize the errors on the SHAPE reactivity values, several data sets were obtained for each condition. Pair-wise comparisons performed between all data sets obtained under the same condition usually showed very good correlation (**Supplementary Figure 1)**, and if the correlation of a data set with the other ones was < 0.70 it was discarded. We averaged data from 3 to 6 experiments for ***Complex, AT-2***, and ***ProtK*** conditions (**Supplementary Dataset 1**). For each protein, the averaged SHAPE values were then used to perform pairwise comparison between the different conditions.

### Pr55^Gag^ and its maturation products differently bind and affect RNA structure

As a reference, we first analysed the structure of the 1–600 gRNA in the absence of protein (***NoProt*** condition) (Figure1(c)**, left part**). NoProt SHAPE reactivities (**Supplementary dataset 1**) and the deduced secondary structure model (**Supplementary Figure 2**) correlated very well with previous studies [26,27], indicating that our 1–600 gRNA folds into the native conformation.

**Figure 2.**
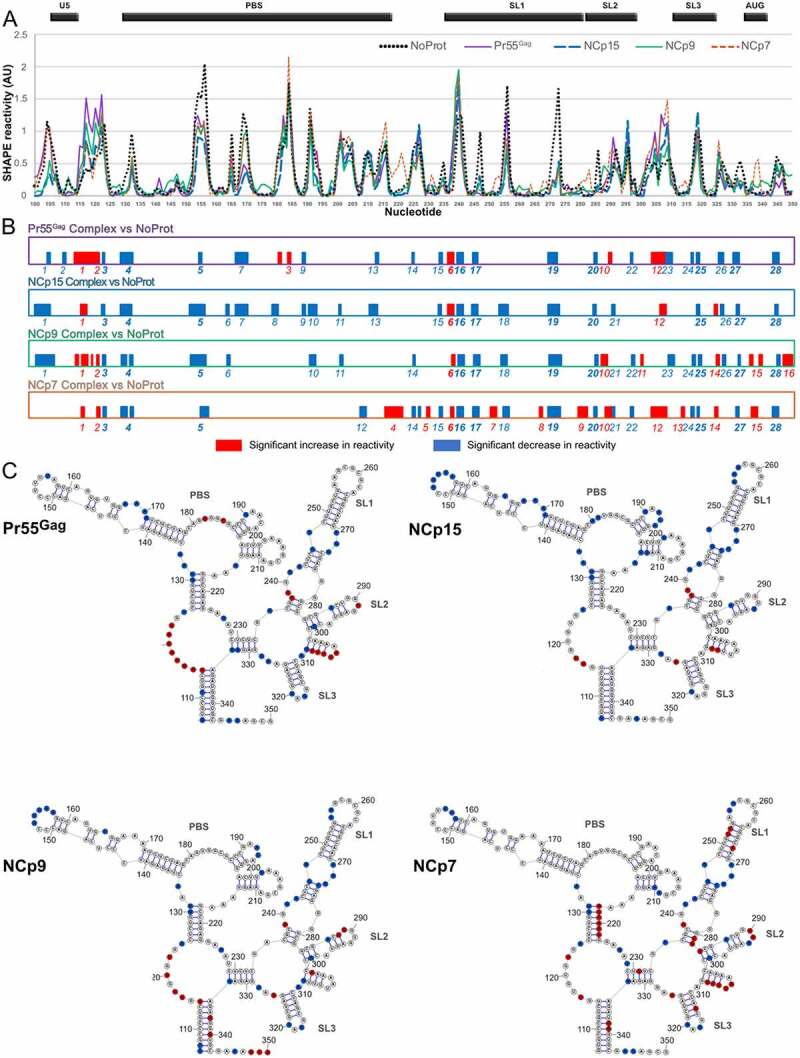
Comparison of the SHAPE reactivity profiles of the 5ʹ region of HIV-1 either alone or in complex with Pr55^Gag^, NCp15, NCp9, or NCp7. (a) The SHAPE reactivity profiles of nts 100–350 under *NoProt, Pr55^Gag^ Complex, NCp15 Complex, NCp9 Complex*, and *NCp7 Complex* conditions are overlaid. (b) The significant differences between the *Complex* and *NoProt* conditions are drawn schematically and numbered. Reactivity decreases upon formation of the complexes are indicated by blue bars, while reactivity increases are indicated by red bars. Regions of decreased and increased reactivity upon complex formation are numbered in blue and red, respectively. All differences presented in this panel were statistically significant and considered to be biologically relevant (see Data analysis in the Method section). (c) These differences are plotted on the RNA secondary structure model obtained using the *NoProt* SHAPE reactivities as constraints, using the same colour code. Since the TAR and Poly A structures were not affected by addition of the proteins, they were omitted in the secondary structure models for clarity.

Pr55^Gag^, NCp15, NCp9, and NCp7 complexes with gRNA 1–600 all show SHAPE reactivity profiles that differ from the ***NoProt*** profile, as well as between themselves (Figure 2(a)). Schematic representation of the significant differences between the ***Complex*** and ***NoProt*** profiles (Figure 2(b)) indicates that protein binding mainly resulted in regions of decreased SHAPE reactivity, although regions of increased reactivity were observed with all proteins. While increased reactivity point to regions of gRNA 1–600 destabilized upon protein binding, decreased reactivity can either reflect protein footprints or RNA regions whose structure is stabilized by the protein.

Overall, vast majority of protected (blue) or destabilized (red) regions are common to several proteins, but only one-third (10/28) of protected regions and one amongst 16 destabilized regions are found for all four proteins (Figure 2(a,b)). This indicates that even if some similarities can be found in the binding of Pr55^Gag^ and its maturation products, they each bind to gRNA and affect its structure in different manners. NCp7 distinguishes itself as having the most pronounced destabilizing effect on the RNA structure, while on the contrary NCp15 barely destabilized the RNA structure at all, and both Pr55^Gag^ and NCp9 had intermediate behaviours (Figure 2(b)). The overall nt composition of the protected regions is similar for all proteins, but slightly more U-rich for NCp15 and NCp9 (**Supplementary Figure 3**). This is due to the fact that these two proteins protect longer stretches of nts than Pr55^Gag^ and NCp7, and these stretches contain ~40% of uridines (Table 1).

**Table 1.** Nucleotides protected by Pr55^Gag^ and its maturations products

	PROTECTED NUCLEOTIDES								
PROTEIN			*Stretch length (nts)*						
			*1*	*2*	*3*	*4*	*5*	*6*	**Total**
		A	4	5	1	6	/	/	
Pr55^Gag^	Nature	C	1	/	/	/	/	/	
		G	6	7	2	1	/	/	
		U	1	/	/	1	/	/	
	**Total**		**12**	**12**	**3**	**8**	/	/	**35**
	Nature	A	3	2	5	7	1	/	
		C	/	/	1	1	/	/	
NCp15		G	7	4	2	3	3	/	
		U	1	/	1	1	6	/	
	**Total**		**11**	**6**	**9**	**12**	**10**	/	**48**
		A	3	5	/	1	1	/	
NCp9	Nature	C	/	1	/	1	/	/	
		G	8	5	/	2	/	3	
		U	1	1	/	/	4	3	
	**Total**		**12**	**12**	/	**4**	**5**	**6**	**39**
		A	4	5	1	1	/	/	
NCp7	ature	C	/	/	/	1	/	/	
		G	6	6	1	2	/	/	
		U	1	1	1	/	/	/	
	**Total**		**11**	**12**	**3**	**4**	/	/	**30**

The total number of nts protected by each protein, as well as the number of nts involved in 1, 2, 3, 4, 5 or 6 protected nt stretches is indicated; the nt composition of these stretches is also reported. U-rich 5 and 6 nt stretches protected by NCp15 and NCp9 are highlighted in pink.

In order to allow a better comparison between Pr55^Gag^ and its maturation products, we plotted the differences observed between the SHAPE profiles under the ***Complex*** and ***NoProt*** conditions on the RNA secondary structure model that best fits the SHAPE reactivity of the ***NoProt*** condition (Figure 2(c)). While protected regions are observed all along the studied region of HIV-1 gRNA, the vast majority of protected regions that were common to all four proteins are located between nts 235 to 333 (Figure 2(b)). This region contains all determinants required for optimal Pr55^Gag^ binding [41] and is usually considered as containing the main HIV-1 packaging signals [42,43,45,57] and is therefore often referred to as the Psi region. Within this region, most common protections were observed in the internal loops of the extended SL1 (Figure 2(c)), which constitutes a Pr55^Gag^ primary binding site [30,58]. An additional protection was observed in the SL1 apical loop (nts 255–257), which mediates HIV-1 gRNA dimerization [49], but only after the Gag precursor underwent the first proteolytic maturation (Figure 2(b,c)). The basal part of SL1 also contains the only region of gRNA 1–600 that is destabilized by the four proteins. (Figure 2(b,c)). Furthermore, single nt protections common to all proteins were also observed in SL2 and SL3, and in the GA-rich region (nts 328–333). Besides, protections common to all proteins also included 4 nts in the basal part of the PBS domain (nts 130–133) (Figure 2(c)).

Most regions destabilized by NCp7 were concentrated in the Psi region. NCp7 uniquely destabilizes the apical and basal stems of SL1 (Figure 2(b,c)), and this may promote the transition from a kissing complex to an extended duplex form of RNA dimer [50,59,60], which has been proposed to correspond to the stabilization of the gRNA dimer observed during the last steps of the viral particle maturation [5,6,16]. NCp7 also destabilized a stretch of four uridines (nts 305–308) that can form an unstable helix located between SL2 and SL3 (Figure 2(c)). Alternatively, these nucleotides might be rearranged into an extended form of SL3. Of note, one study recently proposed that unwinding of this unstable SL3 extension may be crucial for gRNA packaging [34]. Given that this model was based on experiments with mature NCp7 protein, it is interesting to point out that this uridine stretch is strongly destabilized both by NCp7 and Pr55^Gag^, but not by NCp15 or NCp9 (Figure 2(b,c)).

Outside of Psi, we observed that NCp7, but none of the other proteins included in this study, strongly destabilized the 3ʹ strand of the basal stem of the PBS domain (nts 217–223) (Figure 2(b,c)). The 5ʹ strand of this helix contains the primer activation signal (PAS) that is complementary to the TYC arm of the reverse transcription primer tRNA^Lys,3^ and has been proposed to play a key role in the initiation of reverse transcription [39,40]. Additionally, NCp15 protects several stretches of nts in the PBS domain (Figure 2(c)). Of these, the A-rich internal loop (nts 168–171) is complementary to and interacts with the anticodon loop of tRNA^Lys,3^ [36–38]. Altogether, these results suggest that the unique properties of NCp15 and NCp7 stabilize the reverse transcription initiation complex during maturation of the viral particles [61].

### The zinc fingers in the nucleocapsid domain differently interact with HIV-1 gRNA in Pr55Gag and its maturation products

**Figure 3.**
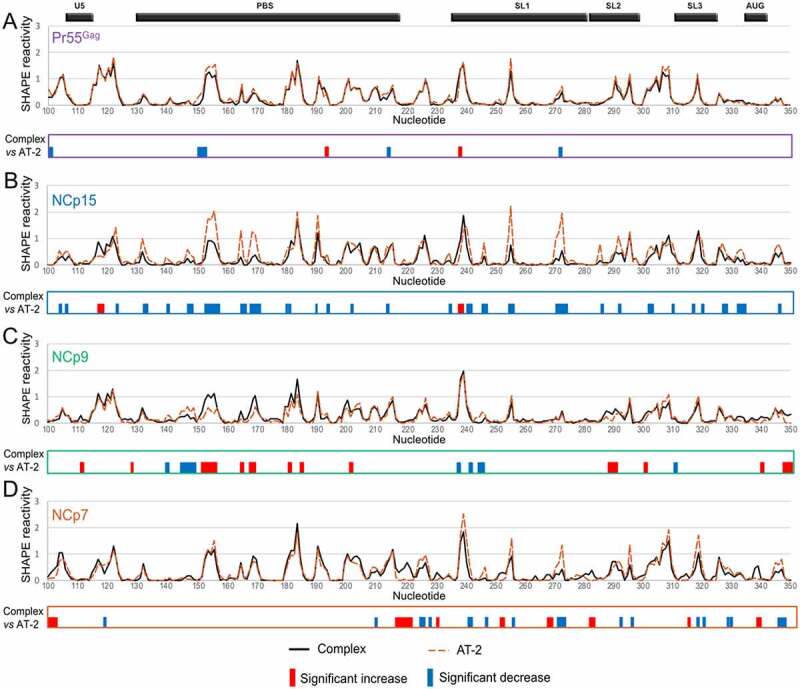
Effect of AT-2 on the RNA:Pr55^Gag^ (a), RNA:NCp15 (b), RNA:NCp9 (c) and RNA:NCp7 (d) complexes. In each panel, the *Complex* and *AT-2* SHAPE reactivity profiles are overlaid in the upper part, whereas the significant differences between the profiles are drawn in the lower part. All differences presented in the lower parts were statistically significant and considered to be biologically relevant (see Data analysis in the Method section). Reactivity increases upon AT-2 treatment are represented by blue bars, while reactivity decreases are indicated by red bars.

Since Pr55^Gag^ and its maturation products were found to bind gRNA 1–600 and to affect its structure in a protein-specific manner, we next asked whether the two conserved zinc fingers (Figure 1(a)) also play specific protein-dependent roles in RNA binding. To this aim, we treated the pre-formed gRNA 1–600:protein complexes with AT-2, which is able to eject the Zn^2+^ ions from the CCHC-type zinc fingers [26], and compared the SHAPE reactivity values obtained in the ***Complex*** condition to those obtained in the ***AT-2*** condition (Figure 3).

On the one hand, AT-2 treatment of the Pr55^Gag^:gRNA 1–600 complex had minimal effect on SHAPE reactivities, suggesting that once the complex is formed, the zinc fingers are not required to maintain the interaction with RNA (Figure 3(a)). Similarly, AT-2 had fairly limited effects on the NCp9:RNA complex, albeit more differences were observed between the ***Complex*** and ***AT-2*** conditions than in the case of the full-length Gag precursor, including a few reactivity decreases upon AT-2 treatment (Figure 3(c)). On the other hand, AT-2 treatment increased the SHAPE reactivity of gRNA 1–600 in complex with NCp15 in numerous regions (Figure 3(b)). Indeed, for NCp15, the SHAPE reactivity differences between the ***Complex*** and ***AT-2*** conditions closely mimicked those observed between the ***Complex*** and ***NoProt*** conditions (compare Figures 2(b) and 3(b)), indicating that the zinc fingers play a crucial role in maintaining the NCp15:RNA complex. A similar observation can also be made for most NCp7 binding sites. With NCp7, not only most of the reactivity increases, but also most of the reactivity decreases were similar when comparing the ***AT-2*** and ***Complex*** conditions or the ***NoProt*** and ***Complex*** conditions (compare Figures 2(b) and 3(d)).

**Figure 4.**
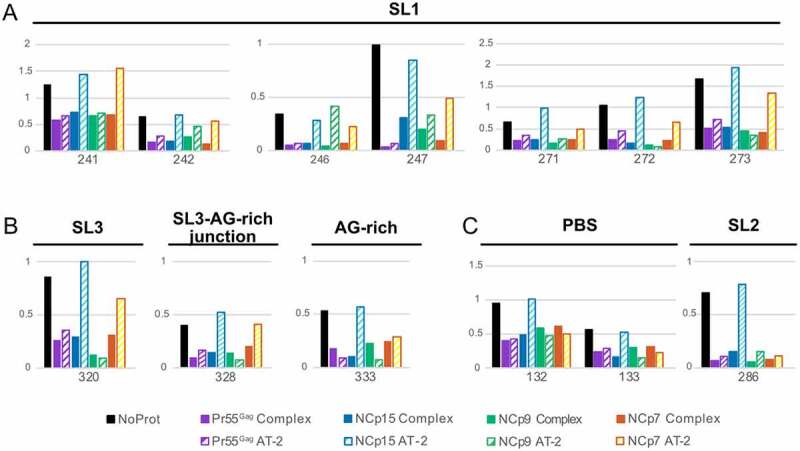
Effects of AT-2 on protected nucleotides located in SL1 (a), SL3, the AG-rich region and the junction between them (b), and PBS and SL2 regions (c). For each nt the SHAPE reactivity in the *NoProt* condition and in the *Complex* and *AT-2* conditions for each of the four proteins are compared.

To obtain a clearer picture of the role of the zinc fingers, we took a closer look at the nts located in the Psi region whose reactivity was significantly affected by addition of all four proteins (Figure 4). For nts in SL1 (*i.e*. nts 241–242, 246–247, 271–273) addition of AT-2 to the Pr55^Gag^:RNA complex (***Pr55^Gag^ AT-2*** condition) had little or no effect on the SHAPE reactivity of the complex, while treatment of the NCp15:RNA complex with AT-2 (***NCp15 AT-2*** condition) increased the SHAPE reactivity to levels similar to those observed in the absence of protein (***NoProt*** condition) (Figure 4(a)). Although the effects of AT-2 on the NCp9:RNA and NCp7:RNA complexes (***NCp9 AT-2*** and ***NCp7 AT-2*** conditions, respectively) were less clear-cut, distinct trends could be observed. AT-2 usually had limited effect on the SHAPE reactivity of the NCp9:RNA complexes (albeit G246 is a noticeable exception), while AT-2 increased the reactivity of the NCp7:RNA complexes at most positions, without always reaching the same reactivity level as in the ***NoProt*** condition (Figure 4(a)). Similar AT-2 effects were observed at nts located in SL3 (nt 320), in the junction between SL3 and the CU:GA interaction (nt 328), and in the GA-rich sequence itself (nt 333) (Figure 4(b)). In the PBS domain (nts 132–133) and SL2 (nt 286), AT-2 retained its destabilizing effect on the NCp15:RNA complexes, but not on the NCp7:RNA complexes (Figure 4(c)), suggesting that NCp7 differently binds to the gRNA regions that are important for packaging and regions that are not. Besides, AT-2 had no effect on the Pr55^Gag^:RNA and NCp9:RNA complexes located in the PBS and SL2 domain (Figure 4(c)), as also observed in the other regions of gRNA 1–600 (Figure 4(a,b)).

### Pr55^Gag^ and its maturation products progressively refold the gRNA structure

**Figure 5.**
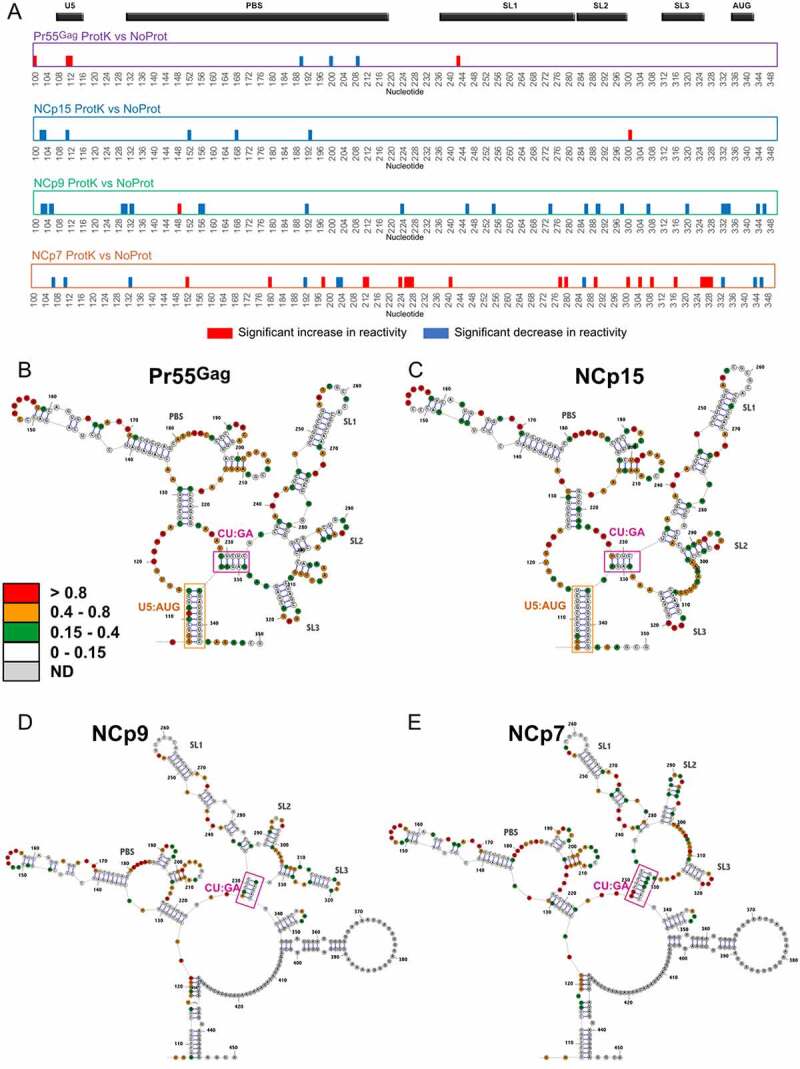
RNA chaperone activity of Pr55^Gag^, NCp15, NCp9, and NCp7 on the gRNA 1–600 structure. (a) The significant differences between the *ProtK* and *NoProt* SHAPE reactivity profiles are represented schematically for each protein. Reactivity increases in the *ProtK* conditions relative to the *NoProt* conditions are represented by red bars, while reactivity decreases are indicated by blue bars. All differences presented in this panel were statistically significant and considered to be biologically relevant (see Data analysis in the Method section). (b-e) Most stable secondary structure models of the 5ʹ region of HIV-1 gRNA obtained using the *Pr55^Gag^ ProtK* (b), *NCp15 ProtK* (c), *NCp9 ProtK (d)*, and *NCp7 ProtK* (e) SHAPE values as constrains. The SHAPE reactivity values are reported on the structures and colour-coded as indicated in the insert. The U5:AUG (when existing) and the CU:GA interactions are indicated in Orange and purple, respectively. Since the TAR and Poly A domains were conserved in all structures, they were omitted for clarity.

Addition of Pr55^Gag^ or its maturation products resulted in numerous protein-specific protections as well as destabilization of gRNA 1–600 (Figure 2), and we next asked whether these effects were transient (*i.e*. if they required the protein to be present in order to be observed) or if they were permanent (*i.e*. if they would persist after removing the protein). Permanent effects on the RNA structure are the hallmark of an RNA chaperone activity, which has been documented for Pr55^Gag^ and its maturation products [13], particularly NCp7 [14,15]. To this aim, we compared, for each protein, the SHAPE reactivity profiles in the ***Complex, ProtK*** and ***NoProt*** conditions (**Supplementary Figure 4**). Indeed, the ***ProtK*** SHAPE profiles were far more similar to the ***NoProt*** profile than to the ***Complex*** profiles, indicating that most of the effects of the proteins on the gRNA structure were transient. Nevertheless, while the differences between the ***ProtK*** and ***NoProt*** profiles were quite limited in the case of Pr55^Gag^ and NCp15, they were more significant with NCp9 and NCp7 (**Supplementary Figure 4** and Figure 5(a)), indicating that the Pr55^Gag^ maturation products that are produced later in the viral maturation process have a more pronounced RNA chaperone activity. However, these two proteins have strikingly different effects on the gRNA structure: while the NCp9 ***ProtK*** profile mainly showed patches of nts whose reactivity decreased compared to the initial ***NoProt*** structure, the NCp7 ***ProtK*** profiles showed equivalent amounts of nt patches with increased and decreased reactivity compared to the ***NoProt*** Structure (Figure 5(a)).

We next took advantage of the ***ProtK*** data to analyse the effects of Pr55^Gag^ and its maturation products on the gRNA secondary structure. In contrast to the ***Complex*** data sets, where SHAPE reactivities are modulated by protein binding, SHAPE reactivites in ***ProtK*** data sets can be used as constraints in RNA folding algorithms to obtain information on gRNA secondary structure. The first (i.e. the most stable) structure predicted when incorporating the ***ProtK*** SHAPE reactivities as constraints are shown for each protein in Figure 5(b-e). All structures presented an identical folding of the TAR and polyA domains, which were therefore omitted for clarity. Considering the limited differences between the ***NoProt*** and Pr55^Gag^ ***ProtK*** SHAPE (Figure 5(a)), it is not very surprising that the secondary structure that best fits the experimental data obtained under these two conditions is identical (compare **Supplementary Figure 2** and Figure 5(b)). Incubation with NCp15 had subtle effects on gRNA 1–600 once it was removed (compare Figure 5(b,c)). The metastable stem-loop located between SL2 and SL3 was destabilized, resulting in its complete unfolding. Besides, the basal part of SL2 was also unwound and the entire SL2 remodelled, and a concomitant extension of SL1 was observed. This in turn shifted the register of the interacting CU-rich and GA-rich sequences by two nucleotides, U230 interacting with A330 (Figure 5(c)) instead of A332 (Figure 5(b)). By contrast the RNA chaperone activity of NCp9 had a dramatic effect on the HIV-1 RNA structure. SL3 was stabilized, forming the extended hairpin recently proposed to play a key role in gRNA packaging [34], which in turn induced a further register shift of the CU:GA interaction, U230 now interacting with A334 (Figure 5(d)). Importantly, this remodelled CU:GA interaction is not compatible with the U5:AUG interaction that has been proposed in an number of HIV-1 RNA secondary structure models [31,41,62,63] and existed in the ***NoProt, Pr55^Gag^ ProtK*** and ***NCp15 ProtK*** secondary structure models (**Supplementary Figure 2** and Figure 5(b,c)), and hence a reorganization of the long-range interactions that maintain the global RNA structure was observed after incubation with and removal of NCp9 (Figure 5(d)). Of note, in this secondary structure model, region 227–337 of HIV-1 gRNA that contains the core Pr55^Gag^ binding domain [41] and the main packaging signal [42] folds into an independent structural domain (Figure 5(d)), as proposed earlier [46]. In the gRNA 1–600 structure remodelled by NCp9, the U5 region surrounding nt 110 interacts with a region located downstream in the *gag* gene (nts 440–446) (Figure 5(d)), rather than with the *gag* AUG codon (Figure 5(b,c)). This new long-distance interaction is maintained in the structure resulting from the NCp7 RNA chaperone activity, while the CU:GA interaction and SL2 are stabilized at the expense of the basal part of the extended SL1 (Figure 5(e)). Similarly, the extended SL3 that was found in ***NCp9 ProtK*** is also destabilized (Figure 5(e)). Significantly, examination of the Los Alamos HIV sequence database (https://www.hiv.lanl.gov/content/sequence/HIV/mainpage.html) indicates that the sequences forming the CU:GA interaction are highly conserved amongst HIV-1 isolates. Likewise, the two lower stems of the U5:*gag* interaction are also highly conserved, indicating that the structural rearrangements we observed with the NL4.3 isolate are also possible in the other HIV-1 isolates, suggesting they are important for function.

Importantly, the structural changes observed with the different proteins do not reflect differences in the dimerization status of gRNA 1–600. Indeed, gRNA 1–600 was 81 ± 2% dimeric in the absence of protein, and the dimer fraction further increased to 89–95% after incubation with the proteins, without any significant difference between proteins (**Supplementary Figure 5**).

## Discussion

The structural rearrangements of the HIV-1 gRNA during maturation of the viral particles remain unexplored, despite evidence that they are required for infectivity [18,19,23,24]. Indeed, despite recent progresses, chemical probing of viral genomes in infected cells, within viral particles, or after extraction is still restricted to infectious viruses [26–29], whereas immature and partially mature viruses are non-infectious. As an alternative approach, here we performed a systematic comparative study of Pr55^Gag^ and its NC-containing maturation products regarding i) the protein binding sites and their transient structural effects, ii) the importance of the two NC zinc fingers in the stability of the complexes, and iii) the permanent effects of these proteins on the HIV-1 gRNA structure. Even though a few previous studies used structural approaches to identify Gag or/and NCp7 [26,32,34,35] binding sites *in vitro* [30–34,41] or directly in viral particles [26,35], no systematic comparison of the Pr55^Gag^, NCp15, NCp9, NCp7 binding sites has been reported. Furthermore, several of these studies were performed using fusion proteins [32,33] or Gag∆p6 [31] as a surrogate for full-length Pr55^Gag^. Similarly, AT-2 has only been used to analyse the contribution of the zinc fingers of mature NCp7 [26], and, to the best of our knowledge, the chaperone activity of Pr55^Gag^ and its maturation products on the 5ʹ region of HIV-1 gRNA has never been described, even though others have analysed the *in vitro* RNA chaperone activity of these proteins using model systems [13,15,38,64].

For the part of this study that can be compared to published works, our results fit well with previous data. Of particular interest is the finding that our *in vitro* NCp7 binding analysis identifies the same binding sites as a similar analysis performed on mature viral particles, indicating that our *in vitro* analysis is relevant to the situation prevailing in viral particles [26]. Similarly, our study reveals that NCp7 has a much greater propensity to remodel the HIV-1 gRNA structure than Pr55^Gag^, in agreement with experiments conducted on small model systems [13,15,65].

**Figure 6.**
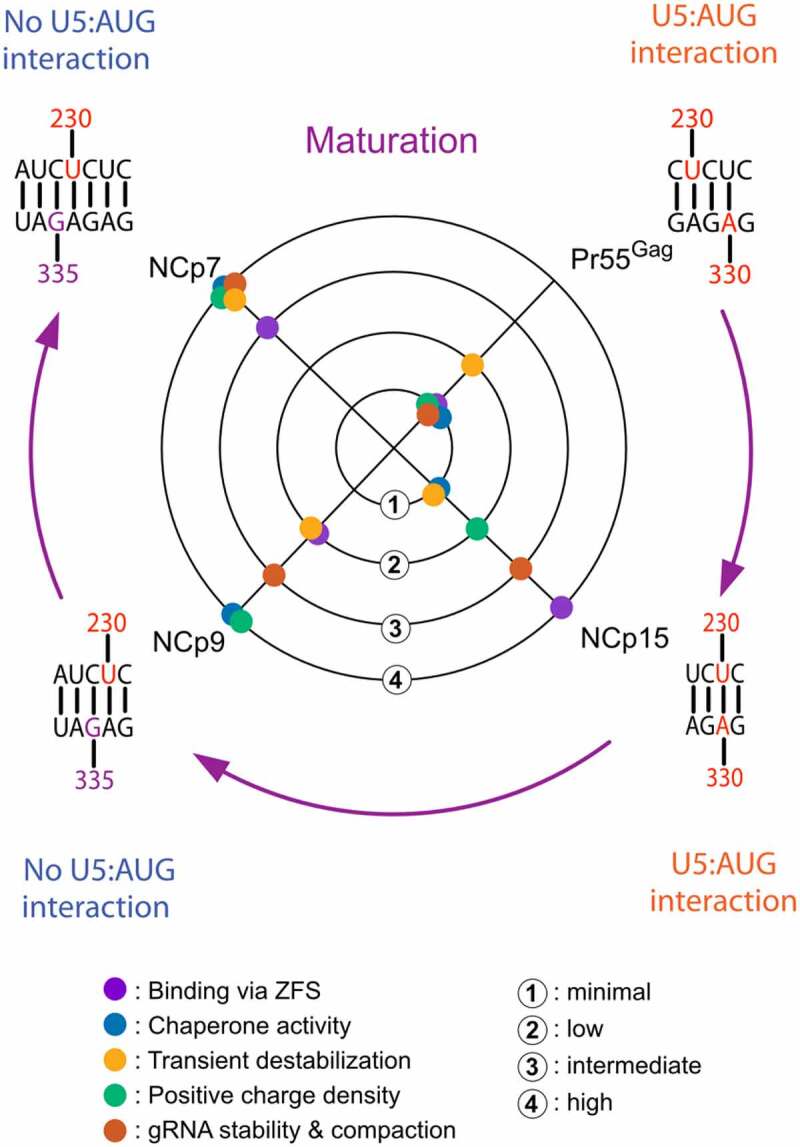
Comparison of Pr55^Gag^ and its maturation products with respect to the contribution of the zinc fingers to RNA binding, RNA chaperone activity, transient destabilization of RNA,overall charge density, and stabilization and compaction of the gRNA dimertransient destabilization of RNA (central part) and evolution of the CU:AG interaction after exposure of the gRNA to Pr55^Gag^ and its maturation products (outer part). Central part: Contribution of the zinc fingers to RNA binding, RNA chaperone activity, and transient destabilization of RNA were assessed semi-quantitatively from results of this study (Figs. 2, 3 & 4, and 5, respectively). Stabilization and compaction of the gRNA dimer were evaluated from published studies [18,19], and the positive charge density of each protein was calculated as the net positive charge at neutral pH divided by the number of amino acids in the protein. The positive charge density is 0.042, 0.107, 0.225 and 0.236 for Pr55^Gag^, NCp15, NCp9, and NCp7, respectively. Outer part: The CU:AG interaction resulting from exposure to NCp9 and NCp7 prevents formation of the U5:AUG interaction.

Overall, our study reveals marked differences in the binding of Pr55^Gag^, NCp15, NCp9, and NCp7 to the 5ʹ region of HIV-1 gRNA: some of the binding sites of these proteins are different and their transient effect on the RNA structure (Figure 2) as well as their chaperone activity differ, leading to different gRNA secondary structures (Figure 5). In addition, the role of the zinc fingers in maintaining the protein:RNA complexes varies from minimal to crucial between Pr55^Gag^ and its NC-containing maturation products (Figures 3 and 4). Strikingly, neither the overall destabilizing effect nor the chaperone activity of Pr55^Gag^ and its maturation products correlates with the role of the zinc fingers in the protein:gRNA complex stabilization (Figure 6**, central part**). Indeed, the zinc fingers have opposite contributions in maintaining the Pr55^Gag^:gRNA and NCp15:gRNA complexes once they are formed, but these two proteins both have minimal chaperone activity and minimal to low transient RNA destabilization ability (Figure 6**, central part**). Remarkably, the effect of AT-2 on Pr55^Gag^ (or NCp15) is the same at all RNA binding sites (Figures 3 and 4), despite the fact that Pr55^Gag^ binds different RNA binding sites with very different affinities [30,41,58]: therefore the opposite effect of AT-2 on Pr55^Gag^ and NCp15 does likely not reflect differences in the affinities of these two proteins for RNA. The fact that AT-2 has no effect on the Pr55^Gag^:RNA complex may seem contradictory with the well-established observation that zinc fingers play a key role in the recognition of retroviral gRNAs by their respective Gag precursors, leading to their specific packaging [66–68]. This contradiction is only apparent, and our data suggest that while the Pr55^Gag^ zinc fingers are required for specific binding to gRNA, they are not required for maintaining the Pr55^Gag^:RNA complex once it is formed. An alternative but less likely interpretation could be that AT-2 is unable to extract the Zn^2+^ ions from the Pr55^Gag^ zinc fingers. Of note, while AT-2 has no significant effect on Pr55^Gag^:RNA complexes and limited effect on NCp9:RNA complexes, it completely destabilizes NCp15:RNA complexes and has also a strong effect on NCp7:RNA complexes. AT-2 thus differently affects the different protein:RNA complexes, and the magnitude of the effect does not correlate with the size of the protein, as would be expected if the protein domains flanking the Zn fingers would provide a steric protection against AT-2. Thus, if the ability of AT-2 to extract Zn^2+^ ions differs amongst the different proteins, this must reflect different binding modes of the Zn fingers in the Pr55^Gag^:RNA and NCp9:RNA complexes on one side and in the NCp15:RNA and NCp7:RNA complexes on the other side.

The RNA chaperone activity of Pr55^Gag^ and its maturation products correlates quite well with the positive charge density of these proteins (calculated as the net positive charge divided by the number of amino acids in the proteins) (Figure 6**, central part**). The primary role of the charge density in the RNA chaperone activity of Pr55^Gag^ and its maturation products is in keeping with the observation that many proteins with disordered positively charged regions, including synthetic polypeptides, display RNA chaperone activity [69–71]. Likewise, the charge density seems to be the main factor governing compaction and stabilization of the gRNA dimer [19] (Figure 6**, central part**).

Pr55^Gag^ and its maturation products mostly bind at identical sites in the Psi region of gRNA (Figure 2), likely reflecting the critical role of the Gag NC domain in the selection and packaging of retroviral gRNA [66–68]. The binding sites of Pr55^Gag^, NCp15, NCp9, and NCp7 in the Psi region exclusively consist in stretches of one to four nts that are highly enriched in purines, and particularly in G residues (73 of the 152 protected nucleotides are G residues (Figure 2 and Table 1)). This observation is in keeping which a recent study indicating that unpaired guanines in the 5ʹ-UTR of HIV-1 gRNA act synergistically to mediate genome packaging [72] and with the central role of guanines for RNA chaperone function [55]. This however does not imply these sites binds Pr55^Gag^, NCp15, NCp9, and NCp7 with the same affinity or that any of these proteins binds all its binding sites with the same affinity [30,73]. The finding that Pr55^Gag^ and its NC-containing maturation products bind at the same sites in the Psi region is however at odds with the widely accepted view that the mature NCp7 completely covers the viral genome in order to protect it [14]. Indeed, many regions remain accessible to the SHAPE probe in the NCp7:gRNA 1–600 complex, and some of these regions may also be accessible to RNases. In light of this observation, the recent finding that reverse transcription takes place in an intact core that is imported into the nucleus and disassembles just before integration appears of particular importance [74,75]. In fact, in contrast with Pr55^Gag^ and its first maturation products, NCp7 extensively destabilizes the Psi region of gRNA (Figure 2). Analysis of mutant HIV-1 blocked at various maturation stages suggests that this destabilization is required for successfully completing reverse transcription [18,19]. In contrast with the Psi region, Pr55^Gag^, NCp15, NCp9, and NCp7 bound differently in the PBS domain (Figure 2). In particular NCp15 and NCp7 show distinctive binding patterns in this domain that may drive the stabilization of the reverse transcription initiation complex [36–40,76] that takes progressively place during maturation of the viral particles [61].

The different RNA chaperone activity of Pr55^Gag^ and its maturation products suggest that the gRNA structure is progressively remodelled inside maturating viral particles. A key region involved in these rearrangements seems to be the CU:GA interaction, with the two interacting strands being progressively shifted as Pr55^Gag^ is replaced by its successive maturation products, eventually causing a dramatic change in the overall gRNA secondary structure (Figures 5 and 6**, outer part**). While SL1, SL2 and SL3 all exist in more or less extended forms (Figure 5), extension of these SLs do not seem to regulate the register shift of the CU:GA interaction since the same forms of SL1, SL2 and SL3 can coexist with different CU:GA interaction registers (compare Figure 5(b,e)). Importantly, the remodelled CU:GA interaction existing after gRNA exposure to NCp9 and NCp7 is not compatible with the U5:AUG interaction that has been proposed in an number of HIV-1 RNA secondary structure models [31,41,62,63] and exists in the ***NoProt, Pr55^Gag^ ProtK*** and ***NCp15 ProtK*** secondary structure models (Compare **Supplementary Figure 2** and Figure 5(b-d)). Of note, in the secondary structure models resulting from the NCp9 and NCp7 RNA chaperone activity, region 227–337 of HIV-1 gRNA that contains the core Pr55^Gag^ binding domain [41] and the main packaging signal [42] folds into an independent structural domain (Figure 5(d)), as proposed in an earlier study [46]. It thus cannot be excluded that, *in vivo*, this gRNA conformer might already exist in producer cells, maybe as a minor one, and be selectively packaged into budding viral particles. In-cell RNA probing methodologies able to detect the coexistence of several RNA conformers, which are currently being developed, should be able to address directly this question. *In vivo* probing could also allow to determine the effect of tRNA^Lys,3^ annealing on the structural rearrangements of gRNA during the maturation process [61]. It would also take into account the fact that there are more Pr55^Gag^ copies in immature particles than NCp7 copies in mature ones [56], whereas we kept the protein concentration constant in all our *in vitro* assays in order to allow a strict comparison between the experimental conditions. Finally, while the gRNA secondary structure models after exposure to NCp9 and NCp7 are very similar (Figures 5(d,e) and 6**, outer part**), the ability of NCp7 to transiently unwind RNA secondary structures (Figures 2 and 6**, central part)**, which is much more pronounced than that of NCp9, is likely crucial for efficient reverse transcription, explaining why infectivity is only acquired after complete maturation of the viral particles.

## Methods

### Protein expression and purification

Expression, purification and characterization of NL4.3 Pr55^Gag^ with an appended C-terminal His_6_-tag was performed as described by McKinstry et al [77]. Recombinant wild-type NCp7, NCp9 and NCp15, respectively 55, 71 and 123 amino acids in length, were expressed and purified as described previously [78–80].

### RNA synthesis

gRNA 1–600 corresponding to the first 600 nts of the wild type NL4.3 HIV-1 gRNA was synthesized by *in vitro* transcription from plasmid pDR4607 [81], after linearization with the *Pvu*II restriction enzyme. Transcription was performed using a MEGAscript T7 Transcription kit (Thermo Fisher Scientific) following the manufacturer’s instructions and purified by exclusion chromatography on a TSKgel G4000SW column as previously described [81]. RNA integrity and purity were confirmed by denaturing polyacrylamide gel electrophoresis.

### Formation of the RNA-protein complexes

In order to form RNA-protein complexes, RNA was folded in a buffer favouring dimerization. Briefly, four pmoles of gRNA 1–600 were denatured in water at 90°C for 2 min then placed on ice for 2 min. The RNA was then incubated at 37°C for 30 min in a 20 µl final volume of 1X Folding Buffer (30 mM HEPES pH 8.0; 300 mM KCl; 5 mM MgCl_2_) supplemented with 40 U RNasin® (Promega) and 1 µg total yeast tRNA. In parallel, 240 pmoles of protein (Pr55^Gag^, NCp15, NCp9 or NCp7) were incubated on ice for 15 min in the 1X Folding Buffer supplemented with 0.01% Triton X-100; 0.01 M DTT and 4 µg BSA in a final volume of 100 µl.

The ribonucleoprotein (RNP) complexes were formed by mixing 20 µl of RNA mixture with 100 µl of protein solution or 100 µl 1X Folding buffer as a control without protein and incubated at 37°C for 30 min then at 0°C for 15 min. The RNP samples were divided in two equal fractions that were treated with a SHAPE reagent (+) or with an equal volume of DMSO as a negative control (-) (see below).

### RNA modification with NMIA

#### Structural effects of protein binding to gRNA 1-600

A 60 µl solution of refolded gRNA 1–600 (***NoProt*** condition) or of protein-RNA complex (***Complex*** condition) was treated with 12 µl of 10 mM NMIA (Sigma-Aldrich) in anhydrous DMSO (Sigma-Aldrich) (+) or with 12 µl anhydrous DMSO (control, -). After 50 min at room temperature, 128 µl Milli-Q water were added and material was precipitated with 3 volumes of ethanol, 1/10 volume of 3 M sodium acetate pH 5.0, 1 µl of glycoBlue (ThermoFisher) for 30 min in a dry ice/ethanol bath and collected by centrifugation at 20,800 g for 30 min at 4°C. The pellets were washed twice with 70% ethanol, dried and resuspended in 15 µl water and 4 µl 5X Folding Buffer. One µl of proteinase K (Roche) was added and the digestion was performed at 37°C for 30 min before adding 79 µl water. The modified RNA was purified by phenol-chloroform extraction (1:1, pH 7.5) and ethanol precipitated as described above. RNA pellets were resuspended in 6 µl Milli-Q water.

#### Effects of AT-2 on the gRNA 1-600-protein complexes

The RNP complexes (60 µl) were treated with 2 µl of 30 mM 2,2ʹ-dithiodipyridine (Aldrithiol, AT-2) (Sigma-Aldrich) in DMSO for 1 h at 37°C. Then, RNA was modified by NMIA using the same protocol as above (***AT-2*** condition).

#### RNA chaperone activity of Pr55^Gag^ and its maturation products

After formation of the RNP complexes as described above, including incubations for 30 min at 37°C and for 15 min at 0°C, the proteins were eliminated by proteinase K treatment prior to RNA modification. The 60 µl mixture was supplemented with 1 µl proteinase K and incubated at 37°C for 30 min. Then, RNA was modified with 12 µl of 10 mM NMIA in anhydrous DMSO or 12 µl DMSO (negative control) and incubated for 50 min at room temperature (***ProtK*** condition). Milli-Q water was added up to 200 µl and the RNA was phenol-chloroform extracted and ethanol precipitated as described above.

### cDNA synthesis and analysis by capillary electrophoresis

RNA samples (6 µl) treated with NMIA (+) or DMSO (-) were mixed with 2 µl of a 1 mM AS primer 1 (5ʹ- AGC TCC CTG CTT GCC CAT ACT A-3ʹ: complementary to nts 436–457 of gRNA 1–600) or AS primer 2 (5ʹ- CTT CTG ATC CTG TCT GAA GG-3ʹ: complementary to nts 536–555 of gRNA 1–600) labelled with Vic (Life Technologies SAS, France). The mixture was heated at 90°C for 2 min and placed on ice for 2 min. After the addition of 2 µl AMV RT Buffer 5x (125 mM Tris-HCl pH 8.3; 250 mM KCl; 10 mM DTT; 25 mM MgCl_2_) and 10 min incubation at room temperature, reverse transcription was performed by adding 2 µl AMV RT Buffer 5x, 6 µl dNTPs 2.5 mM (Invitrogen), 2 U of AMV RT (Life Sciences) and water to 20 µl. Elongation was ensured by incubation 20 min at 42°C followed by 30 min at 50°C. The enzyme was inactivated at 60°C for 10 min. Simultaneously, a sequencing reaction was performed with 2 pmoles of unmodified RNA and 2 µl of a 2 mM AS primer 1 or AS primer 2 labelled with Ned (Life Technologies SAS, France). Reverse transcription was performed as for the SHAPE (+) and (-) elongation reactions except for the reaction mix added which was composed of 6 µl G10 (0.25 mM dGTP, 1 mM dATP, 1 mM dCTP, 1 mM TTP), 2 µl ddGTP at 100 µM, 2 µl AMV RT Buffer 5x and 2 U of AMV RT (Life Sciences).

The reaction volumes were adjusted to 100 µl and cDNAs were phenol-chloroform extracted (Roti-phenol). For each experiment, the modified (+) and unmodified (-) samples were pooled each with a ddG sequencing reaction before ethanol precipitation. The cDNAs were resuspended in 10 µl HiDi Formamide (Applied Biosystem), denatured at 90°C for 5 min, then placed on ice for 5 min and finally centrifuged for 5 min at 6,000 g. The primer extension products were loaded on an ABI 3130XL Genetic Analyser (Applied Biosystem) and the electropherograms were analysed with the QuShape software [82].

#### Data analysis

For pairwise comparisons between the different conditions, we first used deltaSHAPE [83] to identify statistically significant SHAPE differences between two conditions [84]***. DeltaSHAPE performs a modified Z-factor test that identifies nts in the RNA of interest whose SHAPE differences under the two conditions differ by > 1.96 standard deviations of the SHAPE errors under these two conditions (Z-factor > 0), ensuring that the 95% confidence intervals of each measurements do not overlap [83]. However, not all statistically significant differences may be biologically relevant as neither an absolute nor a relative threshold can be used alone to identify the biologically relevant differences between conditions. Using an absolute difference threshold between two SHAPE datasets retrieves false positives (*i.e*. irrelevant differences) when SHAPE reactivities are high in both conditions and may miss biologically relevant differences (false negative) when the SHAPE reactivities are low in both conditions. On the other side, using a relative difference threshold can generate false positive when SHAPE reactivities are low in both conditions, and the difference between reactivity values is not significant. We therefore considered differences between conditions biologically relevant only if the absolute SHAPE reactivities differed by ≥ 0.20 and if the relative difference differed by ≥ 40%.

In order to compare the RNA chaperone effect of Pr55^Gag^ and its maturation products on the gRNA 1–600 structure ‘***NoProt***’ or ‘***ProtK***’ SHAPE reactivity values were used as constraints to fold the RNA secondary structure with the RNAstructure software version 6.0^.^ No other constraints than the SHAPE reactivities were applied to the fold. Based on the RNAstructure data, the structure of naked RNA and deproteinated RNAs were drawn using the Structure Editor graphical tool, a module of the RNAstructure software.

## Supplementary Material

Supplemental Material

## Data Availability

All data are available in the main article or in the supplementary materials.
