# Supplementary material for: Structural maturation of the HIV-1 RNA 5’ untranslated region by Pr55Gag and its maturation products
Source: RNA Biol. 2022 Jan 22;19(1):191–205. doi: 10.1080/15476286.2021.2021677 (PMC8786341; doi:10.1080/15476286.2021.2021677)
Supplement: Supplemental Material [file KRNB_A_2021677_SM1672.zip › supplementary/Gilmer_Mailler_SuppFigs.docx]

**
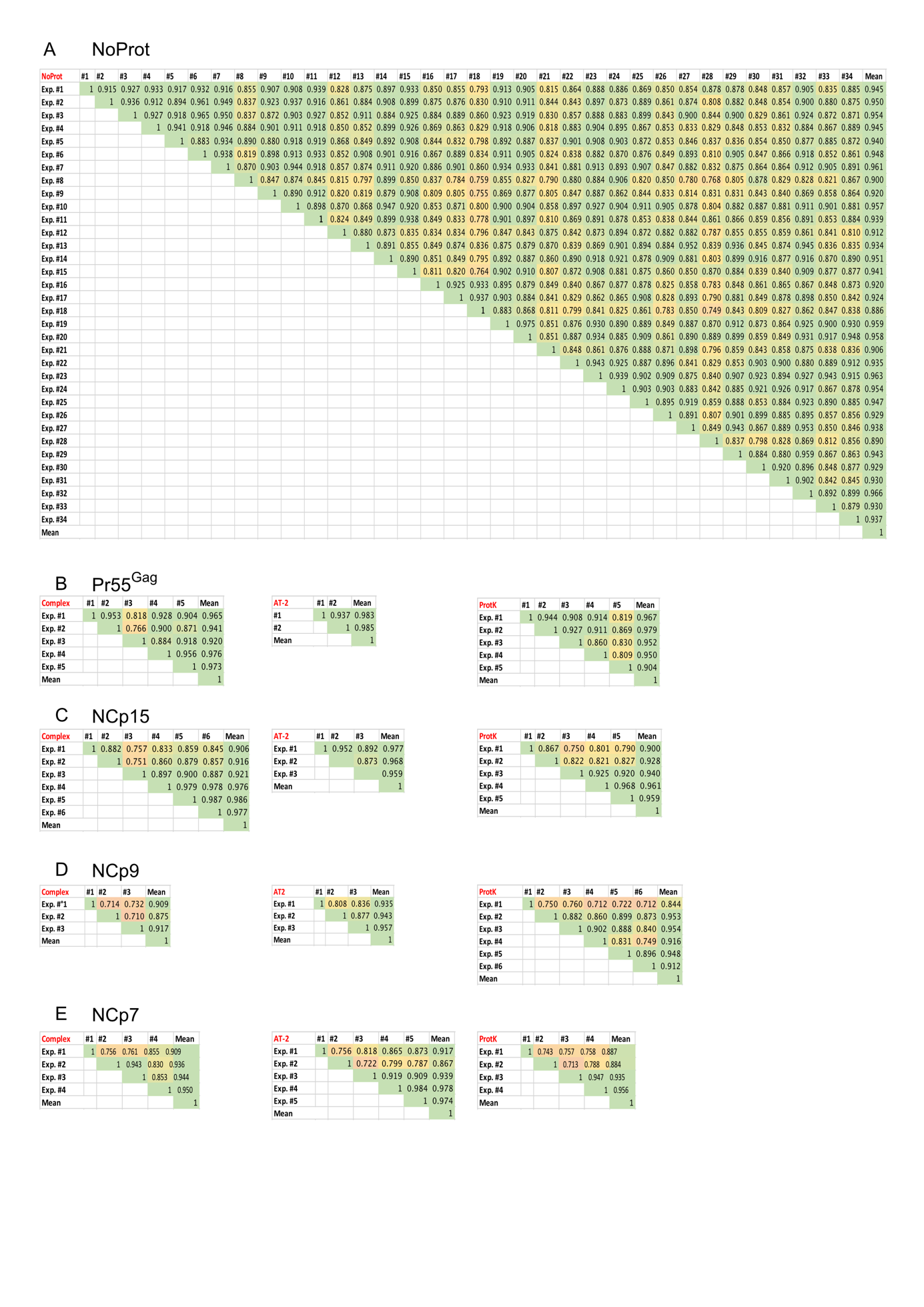
**

**Supplementary Figure 1: Cross-correlation of hSHAPE data sets obtained in the absence of protein (A) and in the presence of Pr55^Gag^ (B), NCp15 (C), NCp9 (D) or NCp7 (E).** For each protein, correlation of the data sets obtained in the ***Complex***, ***AT-2***, and ***ProtK*** conditions are presented. In each table, the columns and lines correspond to individual replicates, except for the last columns and lines, which correspond to the mean value of the replicates.

**
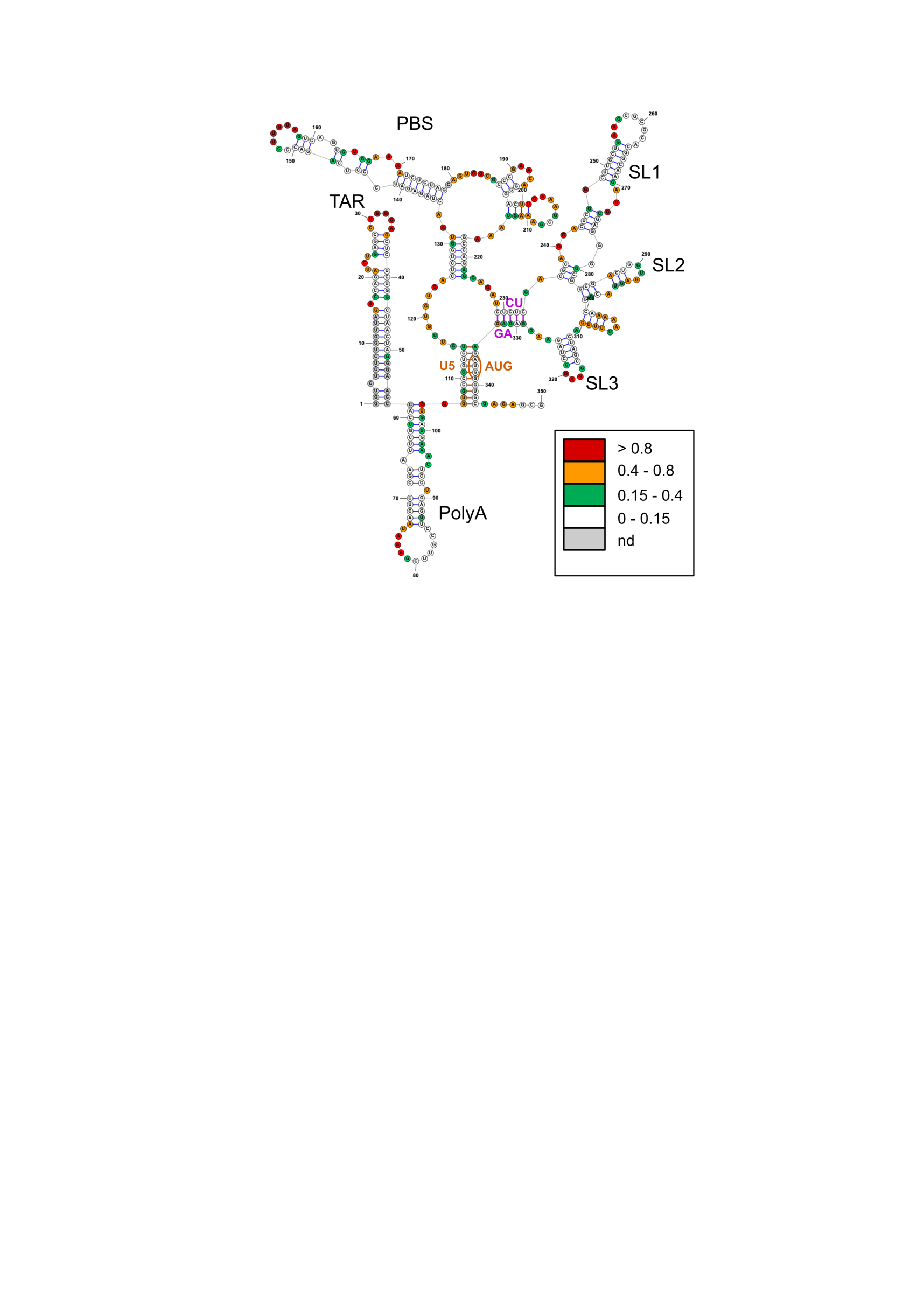
**

**Supplementary Figure 2: Secondary structure model of the 5’ region of the “naked” HIV-1 gRNA.** This structure was obtained using the mean SHAPE reactivity values of the ***NoProt*** condition (mean values of 34 data sets) as constraints for the RNAstructure software (version 6.0). The SHAPE reactivity values are drawn on the structure and color-coded as indicated in the insert. The U5:AUG and CU:GA long distance interactions are indicated in orange and purple, respectively.


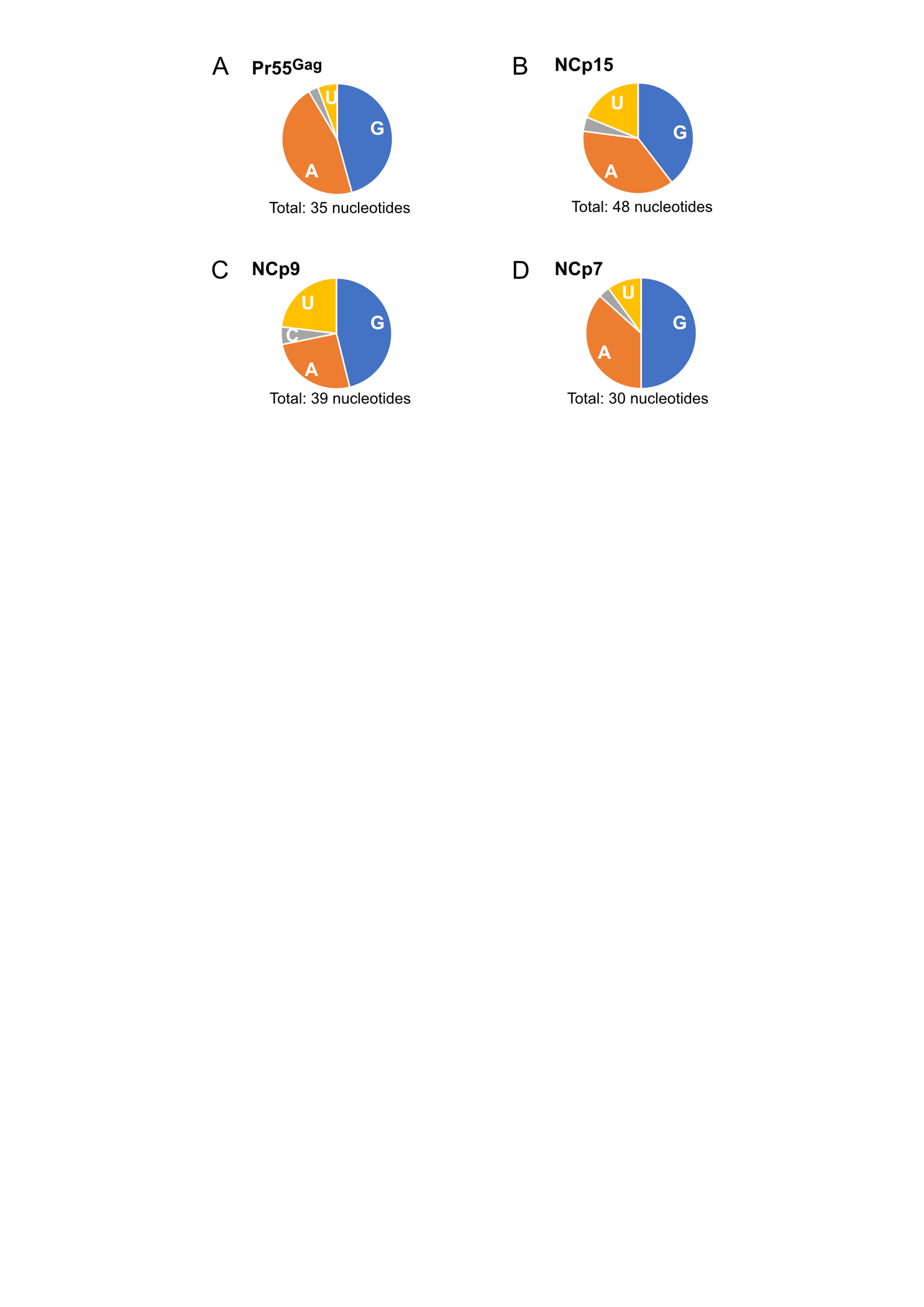


**Supplementary Figure 3: Nucleotide composition of the regions protected by Pr55^Gag^ (A), NCp15 (B), NCp9 (C), and NCp7 (D).**

**
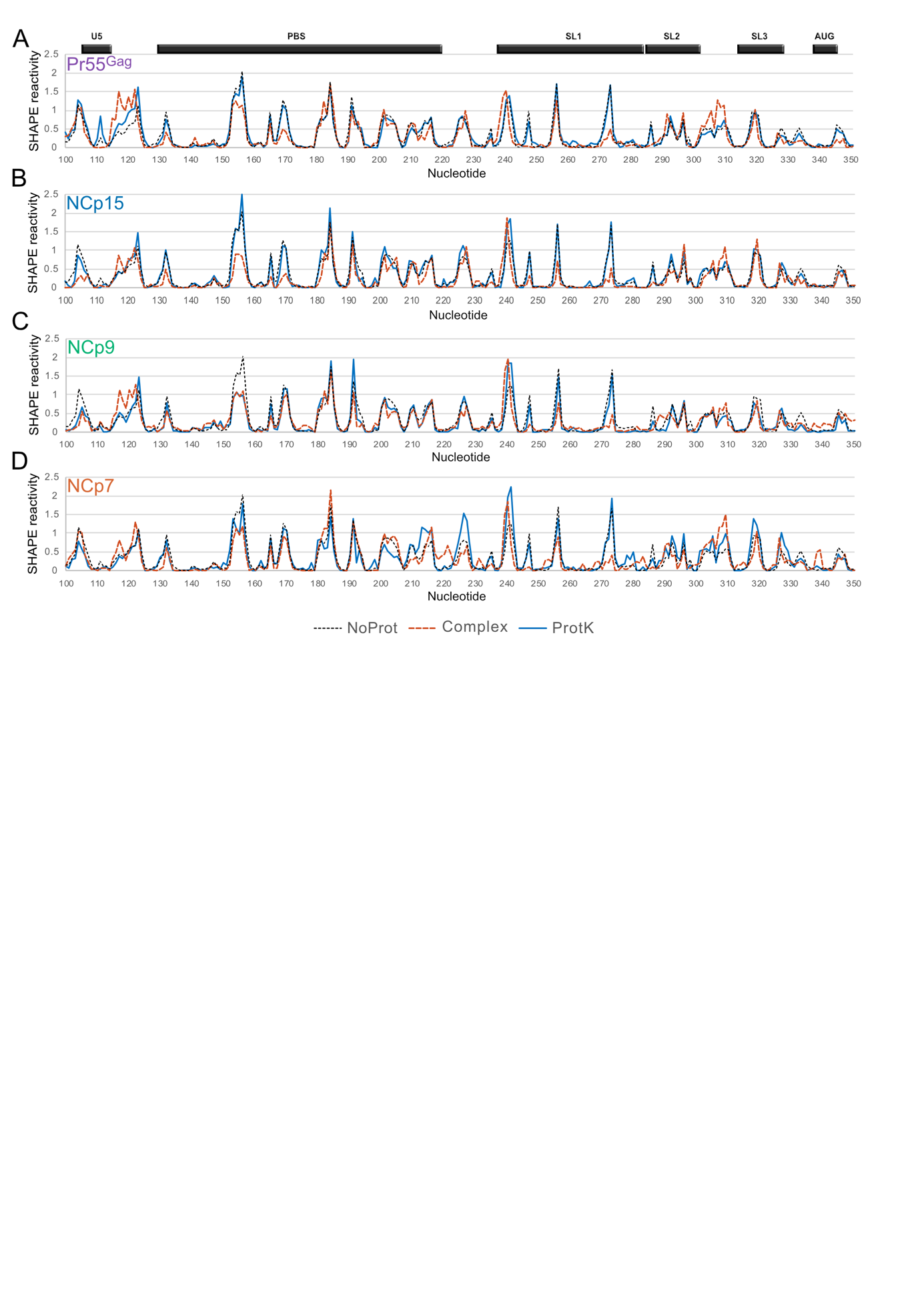
**

**Supplementary Figure 4: SHAPE reactivity profiles under the *NoProt*, *Complex*, and *ProtK* conditions for Pr55^Gag^ (A), NCp15 (B), NCp9 (C), and NCp7 (D).**

**
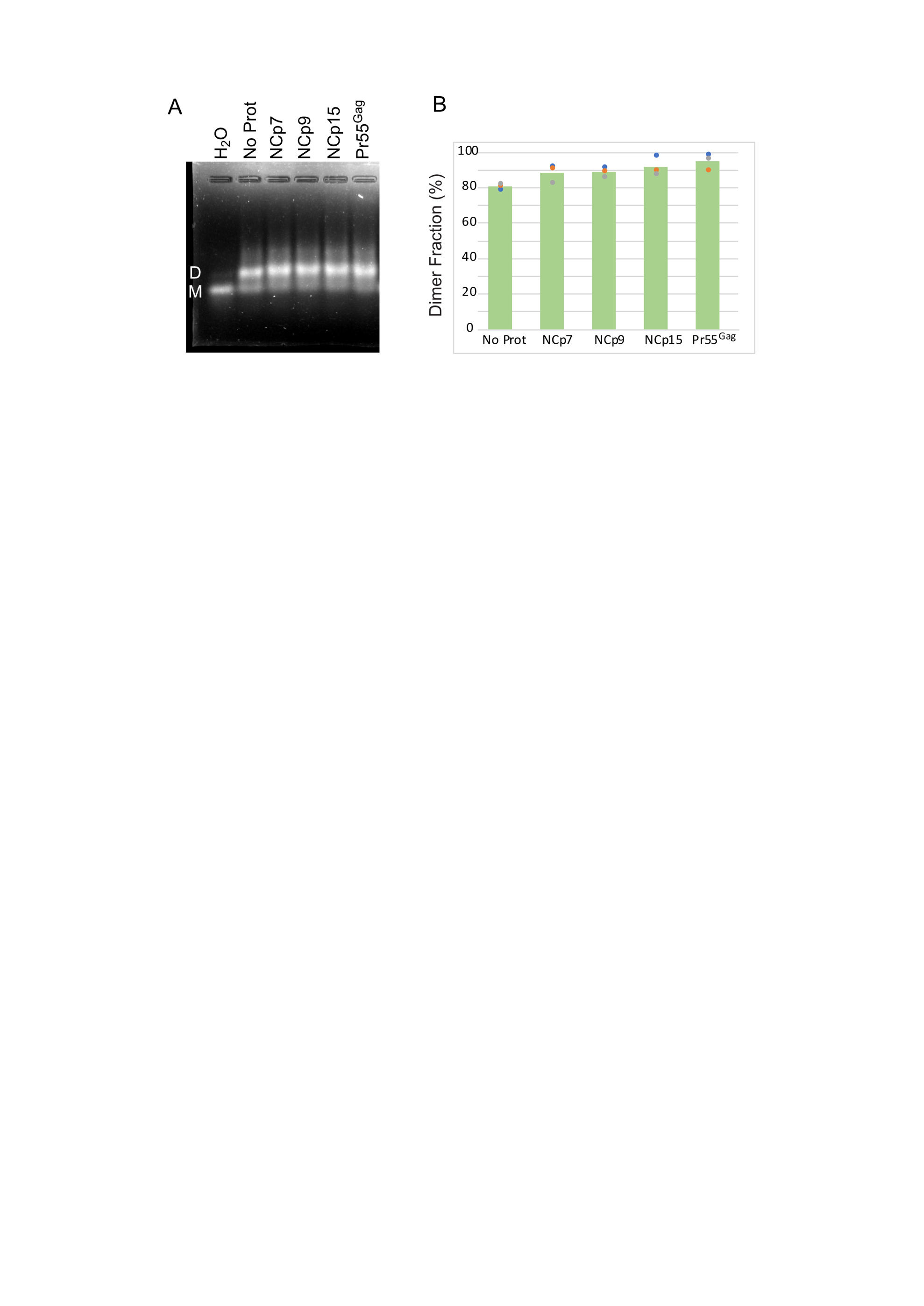
**

**Supplementary Figure 5: Fraction of RNA dimer after incubation without or with protein.** gRNA 1-600 was incubated in the absence of protein or in the presence of Pr55^Gag^, NCp15, NCp9 or NCp7 as described for the ***ProtK*** condition (see material and methods). After removal of the proteins by proteinase K treatment, the samples were analyzed on 1% agarose gels run at 4 °C in Tris-Borate -Magnesium buffer. Experiments were run in triplicates. One of the gels stained with ethidium bromide is shown in **(A)**. M and D stand for dimer and monomer, respectively. RNA dissolved in water was used as a control for monomeric RNA. Quantification of the Dimer Fraction is shown in **(B)**. Fraction Dimer = 100*[D/(M+D)].

**Supplementary Dataset 1**

See accompanying excel file

**Legend to Supplementary Dataset 1**. **Sheet 1**: SHAPE reactivities obtained under the “***NoProt”*** condition. **Sheet 2**: SHAPE reactivities obtained in the presence of Pr55^Gag^ under the ***Complex*** ***ProtK***, and ***AT-2*** conditions. **Sheet 3**: SHAPE reactivities obtained in the presence of NCp15 under the ***Complex*** ***ProtK***, and ***AT-2*** conditions. **Sheet 3**: SHAPE reactivities obtained in the presence of NCp9 under the ***Complex*** ***ProtK***, and ***AT-2*** conditions. **Sheet 4**: SHAPE reactivities obtained in the presence of NCp7 under the ***Complex*** ***ProtK***, and ***AT-2*** conditions. On each sheet, the first column corresponds to the nucleotide position, and for each condition the following columns correspond to different replicates, except the last column, which corresponds to the mean of all replicates obtained under the specified condition. **Sheet 5** summarizes the mean SHAPE values obtained without protein (***NoProt***) and with Pr55^Gag^, NCp15.
